# *In vitro* activity and clinical use of cefiderocol against *Achromobacter xylosoxidans* (Review)

**DOI:** 10.3892/br.2026.2185

**Published:** 2026-08-10

**Authors:** Federico Giovagnorio, Agnese Colpani, Andrea De Vito, Andrea Marino, Giordano Madeddu, Giuseppe Nunnari, Stefano Di Bella, Nicholas Geremia

**Affiliations:** 1 Department of Infectious Diseases, Azienda Sanitaria Friuli Occidentale Santa Maria degli Angeli Hospital of Pordenone, I-33170 Pordenone, Italy; 2 Unit of Infectious Diseases, Department of Medicine, San Francesco Hospital, I-08100 Nuoro, Italy; 3 Unit of Infectious Diseases, Department of Medicine, Surgery and Pharmacy, University of Sassari, I-07100 Sassari, Italy; 4 Unit of Infectious Diseases, Department of Clinical and Experimental Medicine, Azienta Ospedaliera di Rilievo Nazionale e di Alta Specializzazione Garibaldi Hospital, University of Catania, I-95122 Catania, Italy; 5 Clinical Department of Medical, Surgical and Health Sciences, Trieste University, I-34129 Trieste, Italy; 6 Unit of Infectious Diseases, Department of Clinical Medicine, Ospedale dell'Angelo, I-30174 Venice, Italy; 7 Unit of Infectious Diseases, Department of Clinical Medicine, Ospedale Civile ‘S.S. Giovanni e Paolo’, I-30122 Venice, Italy

**Keywords:** *Achromobacter xylosoxidans*, NFGNB, cefiderocol, antimicrobial agents

## Abstract

*Achromobacter xylosoxidans* is an emerging non-fermenting gram-negative bacillus that is increasingly associated with multidrug-resistant infections, particularly among vulnerable patient populations, such as patients with cystic fibrosis and lung transplant recipients. The intrinsic and acquired resistance mechanisms of *A. xylosoxidans*, including efflux pumps, β-lactamase production and altered membrane permeability, limit the effectiveness of standard antimicrobial regimens. Notably, cefiderocol, a siderophore cephalosporin with activity against resistant gram-negative pathogens, has been considered as a potential therapeutic option for *A. xylosoxidans* infections. In the present narrative review, current evidence on the *in vitro* activity and clinical use of cefiderocol against *A. xylosoxidans* is summarised. A structured literature search was conducted using PubMed, Web of Science and Google Scholar to identify relevant studies reporting susceptibility data and clinical outcomes. In conclusion, available *in vitro* and *in vivo* studies generally support the activity of cefiderocol against *A. xylosoxidans*, although some heterogeneity in susceptibility has been observed.

## 1. Introduction

Non-fermenting gram-negative bacilli (NFGNB) represent a heterogeneous group of pathogens that are clinically challenging and increasingly relevant in medical practice. While *Pseudomonas aeruginosa* and *Acinetobacter baumannii* are the most extensively studied organisms in this group, other less common bacteria are emerging as important causes of healthcare-associated and chronic infections, especially in vulnerable populations (1,2). Among these, *Achromobacter xylosoxidans* has progressively shifted from an organism of limited clinical relevance to a pathogen of growing concern, mainly due to its intrinsic resistance profile, high adaptive capacity, and association with difficult-to-treat infections (3).

Initially considered an environmental microorganism with low pathogenic potential, *A. xylosoxidans* is now increasingly isolated in patients with chronic lung diseases, immunosuppression, indwelling medical devices, and prolonged exposure to the healthcare setting (4). The organism is particularly well recognised in patients with cystic fibrosis (CF), where persistent colonisation has been associated with accelerated decline in lung function, frequent pulmonary exacerbations, and worse post-transplant outcomes (5,6). Outside this population, *A. xylosoxidans* has been reported in a wide range of infections, including bloodstream infections, pneumonia, urinary tract infections, endocarditis, and device-related infections. In these clinical scenarios, infections are frequently characterised by prolonged courses, relapses, and suboptimal outcomes, reflecting both host-related vulnerability and the limited availability of effective antimicrobial options (7-9).

One of the main challenges in managing *A. xylosoxidans* infections is its complex, often unpredictable antimicrobial susceptibility profile. This organism exhibits intrinsic resistance to several antimicrobial classes and can acquire additional resistance mechanisms under antibiotic selective pressure (10,11). Consequently, treatment options are often limited to a few agents, such as trimethoprim-sulfamethoxazole, piperacillin-tazobactam, carbapenems, or fluoroquinolones. However, the clinical use of these drugs is frequently compromised by variable susceptibility rates, toxicity issues, and the rapid emergence of resistance (3,12). In addition to these microbiological difficulties, clinical management is further complicated by *A. xylosoxidans'* ability to persist in hostile environments, form biofilms, and survive prolonged antibiotic exposure. For these reasons, clinicians often rely on combination therapies, prolonged treatment courses, or salvage regimens, which are usually supported by limited clinical evidence (13).

The introduction of novel antimicrobial agents with activity against multidrug-resistant gram-negative bacteria has generated cautious optimism. Cefiderocol, a siderophore cephalosporin characterised by a unique ‘Trojan horse’ mechanism of bacterial cell entry, represents one of the most important recent advances in this field (14). By exploiting bacterial iron uptake systems, cefiderocol can reach high periplasmic concentrations and shows stability against many β-lactamases, including metallo-β-lactamases. To date, cefiderocol has been mainly studied in infections caused by carbapenem-resistant *Enterobacterales* (15,16), *P. aeruginosa*, and *A. baumannii*. However, increasing *in vitro* and clinical evidence suggests that its activity may also extend to other problematic NFGNB, including *A. xylosoxidans (*17-19).

Despite this encouraging data, the role of cefiderocol in the treatment of *A. xylosoxidans* infections remains unclear. Available evidence mainly comprises *in vitro* studies, case reports, small case series, and retrospective cohorts, with considerable heterogeneity in patient characteristics, infection sites, treatment duration, and the use of combination therapy. Moreover, important issues remain unresolved, including the risk of resistance development during therapy, the interpretation of susceptibility testing results, and the optimal positioning of cefiderocol within current treatment strategies. These limitations highlight the need for a critical and structured evaluation of the available data.

This review aims to provide a comprehensive overview of *A. xylosoxidans* microbiological characteristics and infections, and to discuss the potential role of cefiderocol in their management, clarifying the strengths and limitations of this antibiotic as a therapeutic option for *A. xylosoxidans* infections, and identifying relevant knowledge gaps that future research should address.

## 2. Microbiology

*Taxonomy. Achromobacter* are considered members of the heterogeneous group of the NFGNB, characterized by their inability to ferment carbohydrates and to derive energy from simple carbohydrates through oxidative pathways (2). *Achromobacter* species are classified as members of the β-proteobacteria (a class within the Pseudomonadota phylum) and belong to the order Burkholderiales. Their taxonomic family is Alcaligenaceae, the same family that comprises the genera *Bordetella* and *Alcaligenes* (20,21). The *Achromobacter* genus currently comprises numerous species widely distributed in the environment, primarily in moist soil, water, and plants, and rarely involved in human pathogenicity (22). In this setting, *A. xylosoxidans* represents the most crucial human pathogen, mainly associated with chronic conditions such as CF or immunosuppression, but also related to nosocomial outbreaks that are often caused by contaminated disinfectant solutions, dialysis fluids, saline solutions and deionised water (22-24).

*Achromobacter* species are frequently misidentified as other common (i.e., *Pseudomonas aeruginosa*, *Stenotrophomonas maltophilia*, *Burkholderia cepacia* complex, *Acinetobacter* spp.), and rare (i.e., *Pandoraea* spp. and *Ralstonia* spp.) NFGNB with conventional methods (i.e., VITEK2) due to biochemical similarities (3). Identification of *Achromobacter* species has become feasible using Matrix-Assisted Laser Desorption/Ionization Time-of-Flight Mass Spectrometry (MALDI-TOF); however, species identification remains challenging with this technique due to limitations in the MALDI-TOF database and limited availability of sequencing methods (2,25). *NrdA* or 16S rRNA gene sequencing, as well as multilocus sequence typing (MLST), represent the two most promising microbiological techniques for identifying the *Achromobacter* family at the species level (22). Regarding sequence-based identification, *nrdA* gene sequencing or MLST, which targets seven housekeeping genes (*nusA*, *rpoB*, *eno*, *gltB*, *lepA*, *nuoL*, and *nrdA*), provides a more accurate tool for species-level identification (26). In contrast, 16S rRNA gene sequencing is insufficient for definitive species discrimination because of the highly conserved nature of the gene (24). With the continuous advancement of sequencing technologies, whole-genome sequencing has become an increasingly valuable and precise method for identifying *Achromobacter* species, with the capability of reclassifying taxa that were historically misclassified (22,24).

### Antimicrobial susceptibility issues

There is also the challenge of defining *Achromobacter*-specific breakpoints and identifying appropriate reference methods for antimicrobial susceptibility testing (AST) of this pathogen. The current Clinical and Laboratory Standards Institute (CLSI) guidelines for AST of *Achromobacter* spp. extrapolate the breakpoint for this pathogen directly from a general non-*Enterobacterales* breakpoint, which is used for all non-fastidious, non-glucose-fermenting gram-negative bacilli. This approach has several significant limitations, including the lack of species-specific breakpoints (27).

One of the greatest problems is that these breakpoints have not been revised since their initial publication, whereas, due to new pharmacokinetic/pharmacodynamic evidence and the emergence of antimicrobial resistance mechanisms, *P. aeruginosa* and *Enterobacterales* have undergone revisions. Consequently, non-*Enterobacterales* breakpoints likely provide suboptimal estimates of susceptibility and warrant reassessment (12). Non-*Enterobacterales* breakpoints pose an intrinsic problem in their application, as only minimum inhibitory concentration (MIC) breakpoints are available for this group, and commercial AST systems exhibit high uncertainty in performance for *Achromobacter* spp.

Thirdly, the non-*Enterobacterales* group includes multiple genera, leading to breakpoints for antibiotics to which *Achromobacter* is intrinsically resistant (e.g., aminoglycosides, cephalosporins except ceftazidime, and aztreonam). These agents may be incorrectly reported as susceptible because of ambiguous breakpoints and uncertainty regarding AST accuracy (12,28). To address this gap, Harris *et al* (12) proposed tentative CLSI M45 MIC and disk diffusion breakpoints for *Achromobacter* spp. for four antimicrobials: meropenem, imipenem, piperacillin-tazobactam, and trimethoprim-sulfamethoxazole.

Unlike CLSI, the European Committee on Antimicrobial Susceptibility Testing has proposed *A. xylosoxidans* specific breakpoints for different antibiotics, including piperacillin-tazobactam (S≤4, R>4), meropenem (S≤1, R>4), and trimethoprim-sulfamethoxazole (S≤0.06, R>0.06) (29).

### Resistance mechanisms

In terms of antimicrobial susceptibility, *Achromobacter xylosoxidans* is intrinsically resistant to most cephalosporins (except ceftazidime), as well as to aztreonam, ertapenem, and aminoglycosides (2,30). At the same time, the clinical approach is strongly influenced by the ability to develop numerous acquired resistance mechanisms.

Antibiotic resistance among *Achromobacter* spp. can be attributed to various mechanisms, including chloramphenicol acetyltransferase, aminoglycoside acetyltransferase, aminoglycoside phosphotransferases, dihydrofolate reductases, mutations in gyrA, ParC, and rRNA methylases, β-lactamases [such as metallo-β-lactamases (MBL)], resistance-nodulation-cell division (RND) efflux pumps, and biofilm formation (2,12,20,31). Many members belonging to the RND efflux pump group are associated with intrinsic resistance. Among them, AxyABM and AxyXY-OprZ are the two most important mechanisms discovered in *A. xylosoxidans* (32). AxyABM can extrude most cephalosporins (except cefuroxime and cefepime), fluoroquinolones, aztreonam and chloramphenicol; instead, AxyXY-OprZ, can affect aminoglycosides, cefepime, fluoroquinolones, tetracyclines, tigecycline and carbapenems (2,20,31). Among β-lactamases, *Achromobacter* species produce a constitutive chromosomal β-lactamase, namely OXA-114(3). Still, in *A. xylosoxidans*, numerous genes for other β-lactamases were found, including *bla*_AXC_, *bla_CTX-M_*, *bla_VEB-1_*, *bla_AmpC_*, *bla_CMY−2_*. *bla_IMP_*, *bla_TMB-1_* and *bla_VIM-4_* (3,12,33,34). OXA-114-like enzymes exhibit strong *in vitro* activity against penicillin G, cephalosporins, piperacillin, and ticarcillin. Nevertheless, the contribution of OXA-114 to phenotypic piperacillin resistance remains unclear, as piperacillin susceptibility is frequently observed phenotypically among OXA-114-positive *A. xylosoxidans* strains (3,20,35). Moreover, tazobactam could positively impact the piperacillin MIC, resulting in a 1-fold decrease in the piperacillin MIC (36). OXA-114-like enzymes minimally hydrolyse ticarcillin and imipenem, while extended-spectrum cephalosporins such as ceftazidime, cefotaxime, and cefepime are not substrates (2,3). The dissemination of acquired β-lactamases, especially MBLs, is worrisome. Aztreonam is the only β-lactam option that MBLs cannot hydrolyse; however, its activity is reduced by the presence of the AxyABM efflux pump. The prevalence of MBLs among carbapenem-resistant *Achromobacter xylosoxidans* isolates remains poorly understood, despite the rising rates of carbapenem resistance worldwide (3).

The resistance mechanisms described are not the only weapons available to *Achromobacter xylosoxidans*; chronic colonisations are permitted by a high capability to survive the host immune system and antibiotic therapies by increasing the efficiency in nutrient acquisition, developing the ability to avoid toxic compounds and to evade immune response by forming biofilm colonies (20).

## 3. Clinical settings of Achromobacter xylosoxidans infection

*A. xylosoxidans* is increasingly recognized as a clinically significant opportunistic pathogen, particularly in healthcare settings and immunocompromised populations (37). The epidemiology and clinical manifestations of *A. xylosoxidans* infections have evolved substantially over the past two decades, reflecting its emergence as an important nosocomial pathogen alongside its established association with specific patient populations and anatomical sites of infection.

### Respiratory tract infections in cystic fibrosis patients

The most extensively characterized clinical setting for *A. xylosoxidans* infections is among individuals with CF, where the organism has emerged as a significant late-stage colonizer (5,38). The global prevalence of *A. xylosoxidans* in CF populations has increased strikingly in recent years, from 2.7% in 2001 to current estimates of 5.4% among nearly 2,100 European CF patients, with some regional centres reporting prevalence rates exceeding 13.9% (39). This upward trend reflects both the organism's environmental prevalence and the changing epidemiology of CF pathogens, driven by prolonged patient survival and broad-spectrum antibiotic use.

CF patients chronically infected with *A. xylosoxidans* consistently exhibit evidence of disease progression and enhanced clinical morbidity compared to uninfected subjects (38). Multiple prospective studies have documented significant declines in forced expiratory volume in 1 sec (FEV_1_). These patients also demonstrate increased frequency of pulmonary exacerbations requiring hospitalization, higher treatment burden with more frequent antibiotic administration, and greater steroid use (38,39). In addition to pulmonary function decline, CF patients with *A. xylosoxidans* infection exhibit lower body mass index and a higher risk of requiring lung transplantation, with increasing adverse events such as augmented mortality (40). The pathophysiological basis for these clinical sequelae has been elucidated through experimental studies demonstrating that *A. xylosoxidans* exhibits robust *in vitro* adherence to polarized CF bronchial epithelial cells, causes significant cytotoxicity, and establishes persistent lung colonization in murine infection models with associated inflammatory consequences including neutrophil influx, enhanced cytokine production, and pulmonary damage (40,41).

### Catheter-associated and healthcare-related bloodstream infections

*A. xylosoxidans* has emerged as an important cause of catheter-related infections in hospitalized patients, representing one of the most common clinical presentations of this organism (42,43). A comprehensive case review identified catheter contamination as the underlying risk factor in 60% of bacteraemia cases, with contaminated intravenous solutions and biofilm formation on central venous catheters representing the principal mechanisms of infection (44). Patients with intravascular devices, including central venous catheters, peripherally inserted central catheters, and peritoneal dialysis catheters, face a significantly higher risk than the general population. In a retrospective analysis of nosocomial *A. xylosoxidans* infections, 48% of clinical isolates were obtained from blood cultures, 80% of affected patients had medical devices in situ at the time of infection, and 87% presented with a recent intensive care unit admission (39,45). The organism's propensity to form biofilms on catheter surfaces creates a protected microenvironment that facilitates persistent colonization and recurrent bacteraemia (46). Notably, intermittent manipulation of catheters, including routine flushing procedures, can dislodge biofilm and precipitate symptomatic bacteraemia, with documented cases of symptomatic infection occurring more than one year following initial contamination exposure (44). The mortality associated with *A. xylosoxidans* bacteraemia has been reported at approximately 15%, emphasizing the clinical significance of this pathogen in hospitalized populations (44). Patients with underlying malignancy represent a particularly high-risk group, as they typically require prolonged indwelling catheters for chemotherapy administration and are rendered immunosuppressed through the combined effects of the neoplasm, cytotoxic therapy, and frequently prolonged corticosteroid administration (47,48).

### Infections in immunocompromised populations

*A. xylosoxidans* infections occur predominantly in severely immunocompromised individuals, including those with hematologic or solid organ malignancies, patients experiencing neutropenia from chemotherapy, recipients of hematopoietic stem cell or solid organ transplants, and individuals with chronic kidney disease. In these patient populations, *A. xylosoxidans* has been documented as causative of bacteraemia, pneumonia, meningitis, urinary tract infections, and various localized infections. An important consideration is that immunocompromised patients frequently harbour multiple comorbidities, with studies documenting that 50-67.5% of patients infected with *A. xylosoxidans* have underlying cardiac disease, malignancy, or recent surgical procedures (39,49). Advanced age, typically defined as 50-65 years or older, has been identified as an independent risk factor, with the highest frequency of infections in older patient cohorts (39).

### Other manifestations

*A. xylosoxidans* has also been documented as causative of a diverse array of infections affecting nearly every organ system. Meningitis represents a particularly serious manifestation (50,51), occurring predominantly in severely immunocompromised patients and increasingly in association with implanted intrathecal devices such as epidural catheter or delivery systems (52). The diagnosis of *A. xylosoxidans* meningitis is frequently delayed due to the organism's rarity and may require molecular confirmation via cerebrospinal fluid analysis.

Prosthetic valve endocarditis caused by *A. xylosoxidans* has emerged as a recognized clinical entity, characterized by difficult diagnosis and treatment (53). Scientific literature highlights the challenges inherent in identifying this organism within biofilm-forming communities on infected valves, as conventional culture and advanced imaging studies, including positron emission tomography and transoesophageal echocardiography, proved insufficient for diagnosis; definitive identification required molecular techniques using fluorescence in situ hybridization combined with 16S rRNA sequencing (54,55). The biofilm-producing capacity of *A. xylosoxidans* within heart valve tissue explains the observed therapeutic failures despite adequate antibiotic treatment and the propensity for relapsing bacteraemia even with appropriate antimicrobial therapy (54,56).

Peritonitis in peritoneal dialysis patients represents an additional important clinical manifestation. While historically considered extremely rare, emerging evidence suggests increasing frequency, with documented cases of recurrent peritonitis related to biofilm formation on peritoneal dialysis catheters (57). Removal of the infected catheter remains essential for resolving the infection, as systemic antimicrobial therapy alone is inadequate due to biofilm-mediated protection.

Additional reported manifestations include urinary tract infections associated with indwelling urinary catheters (58,59), chronic otitis media and otitis externa (60,61) (the original reported site of isolation in the initial 1971 description of the organism), and wound infections in post-surgical patients (43). Hospital-acquired pneumonia in mechanically ventilated patients represents another recognized presentation, typically occurring in elderly patients with significant underlying comorbidities (62,63).

From a clinical point of view, *Achromobacter* spp. poses significant diagnostic and therapeutic challenges due to their opportunistic nature, frequent involvement in device-associated and chronic infections, and intrinsic multidrug resistance. Clinical management is further complicated by delayed recognition, biofilm formation, and the lack of standardized treatment strategies, particularly in vulnerable patient populations.

## 4. Cefiderocol activity *in vitro*

Although limited, studies on cefiderocol activity against *Achromobacter* spp. are increasing in the literature, and its use in real-world settings is well established. Tunney and colleagues (64) tested 74 strains of *Achromobacter* species, of which 69 were *A. xylosoxidans*, and reported a cefiderocol MIC <2 mg/l in 87.8% of the strains. The Bruker UMIC cefiderocol assay on Sensititre gram-negative EUMDRXXF AST plate was used in this study. Even more encouraging results, although on a smaller sample size, were reported by Oueslati *et al* (65) Among 12 strains of *A. xylosoxidans*, two were carbapenem-resistant; all strains were susceptible to cefiderocol.

As previously discussed, CF patients represent an exceptionally challenging population to treat for *Achromobacter xylosoxidans* infection. The study by Beauruelle *et al* (66) focused on 23 isolates from CF patients. Cefiderocol retained susceptibility in 91% of strains, showing the highest susceptibility rate in this study, probably due to the selective pressure of numerous antibiotic courses to which this specific population is exposed, and to which cefiderocol may be less susceptible than other antibiotics.

Larger studies addressing *Achromobacter* spp. susceptibility to cefiderocol have been published by Takemura *et al* (17) and Jean-Pierre *et al* (19). Takemura *et al* (17) examined 334 strains of *Achromobacter* species, of which 311 were *A. xylosoxidans*. MIC >2 mg/l was observed only in 11 strains. Of note, cefiderocol sensitivity was retained in 48/52 strains non-susceptible to carbapenems. Six strains showed MICs of 16-64 mg/l; whole-genome sequencing revealed OXA-114- or OXA-364-based β-lactamases (17).

Recently, Jean-Pierre *et al* (19) reported *in vitro* data on 230 strains of *Achromobacter* spp., of which 164 were assigned to *A. xylosoxidans*. Most isolates were susceptible to cefiderocol. Only two strains (0.9%) had MICs >2 mg/l; both were *A. xylosoxidans* and were collected from CF patients. Among these two strains, one was multidrug resistant (MDR; thus resistant to meropenem, trimethoprim-sulfamethoxazole, and piperacillin-tazobactam), and the other retained susceptibility at increased exposure to meropenem. Of the eight MDR strains included, the other seven retained susceptibility to cefiderocol. Of the 27 strains non-susceptible to meropenem, 26 remained susceptible to cefiderocol. Of note, *A. xylosoxidans* strains displayed statistically significantly lower MIC to cefiderocol than the other strains.

All available studies use the BMD reference method, which is based on iron-depleted cation-adjusted Mueller-Hinton broth. A study by Jean-Pierre *et al* (67) explored possible alternatives to the recommended method, which is complex and time-consuming. However, none of the so far available alternatives (disk diffusion and other broth dilution methods) met an acceptable level of agreement.

## 5. Clinical data on cefiderocol

As outlined in the previous paragraph, these gram-negative pathogens, are intrinsically resistant to multiple antimicrobial classes, including most cephalosporins, aztreonam, aminoglycosides, and frequently carbapenems. Available treatment options are generally restricted to trimethoprim-sulfamethoxazole, piperacillin-tazobactam, or fluoroquinolones, each associated with variable efficacy and rising resistance rates (3). As a result, and similarly to *Acinetobacter baumannii*, *Achromobacter* spp. predominantly affects patients with significant underlying conditions. Infections are associated with considerable morbidity and mortality (~30-40%) (18,68), particularly among individuals with CF, solid-organ transplant recipients, and immunocompromised hosts. Within this context, cefiderocol has emerged as a promising salvage therapy. The main clinical studies and real-life experiences are summarised in Table I.

One of the earliest clinical signals was reported by Bodro *et al* (2021) (69), who described two cases of multidrug-resistant gram-negative infections in patients with intravascular devices. One case involved *A. xylosoxidans* in a patient with a left ventricular assist device, successfully treated with cefiderocol in combination with tigecycline and piperacillin-tazobactam, leading to clinical resolution without device removal (69). Subsequently, La Bella *et al* (70) reported a patient with non-Hodgkin lymphoma who developed *A. xylosoxidans* aortic endocarditis. Combination therapy with cefiderocol (MIC 1 µg/ml), trimethoprim-sulfamethoxazole, and fosfomycin achieved clinical cure. Notably, the authors provided pharmacodynamic evidence of marked increases in serum bactericidal activity after cefiderocol initiation, from undetectable levels at baseline to titres of 1:8 at trough and 1:32 at peak concentrations (70).

Regarding CF patients, *A. xylosoxidans* is isolated in 3-8% of CF cases and is associated with accelerated lung function decline and poor post-transplant outcomes. Warner *et al* (71) described a compassionate-use series of eight patients (adults and children) with extensively drug-resistant *A. xylosoxidans*. Across twelve cefiderocol treatment courses (median duration 14 days, range 7-21), clinical response was achieved in 11 of 12 episodes (91.6%). However, microbiological relapse, defined as re-isolation of *Achromobacter xylosoxidans* within 180 days, occurred in 11 of 12 courses (91.6%). Pretreatment resistance to cefiderocol was documented in 3 of 8 patients (37.5%), yet clinical improvement was still observed, highlighting the partial dissociation between *in vitro* MICs and clinical outcomes in CF. Combination therapy, most frequently with piperacillin-tazobactam, ceftazidime-avibactam, colistin, or tigecycline, was commonly employed to mitigate the risk of monotherapy failure (71).

An additional paediatric case was reported by Gainey *et al* (72), involving a ten-year-old girl with CF chronically infected by pan-drug-resistant *Achromobacter xylosoxidans*. The patient received a 14-day course of cefiderocol in combination with a patient-specific bacteriophage. Despite *in vitro* resistance (MIC 32 µg/ml), the regimen led to rapid clinical improvement, with FEV_1_ increasing from 33 to 60% within 12 days. Sputum cultures remained negative at 8 and 16 weeks, suggesting that synergistic strategies incorporating phage therapy may provide durable microbiological suppression in otherwise refractory cases (72).

More structured evidence derives from early access and real-world cohorts. In the Spanish PERSEUS program, Torre-Cisneros *et al* (18) analysed 14 patients with infections caused by rare non-fermenting gram-negative bacilli, including 5 cases of *Achromobacter* spp. Sites of infection included the respiratory tract (n=2), urinary tract (n=2), and bloodstream (n=1, catheter-related). Two patients were transplant recipients. Cefiderocol was used in combination with other agents in two cases, most commonly meropenem, ceftazidime-avibactam, or colistin. Clinical cure was achieved in four of five patients (80%), but 28-day all-cause mortality remained 40%, reflecting the vulnerability of this patient population (18). Similarly, Kirkegaard-Biosca *et al* (68) retrospectively evaluated 34 patients with infections caused by VIM-producing gram-negative bacilli, including 5 cases of *Achromobacter xylosoxidans.* The median duration of cefiderocol therapy was 13 days, clinical failure at day 14 was 15%, and 30-day mortality was 27%. Importantly, one case demonstrated the emergence of resistance during treatment, confirming concerns raised in preclinical studies.

Endocarditis caused by *Achromobacter* spp. is rare but often associated with poor outcomes. Wackernagel *et al* (56) described prosthetic valve endocarditis due to *A. xylosoxidans* following transcatheter aortic valve implantation. The infection was characterized by three months of relapsing bacteraemia, refractory to meropenem and piperacillin-tazobactam. Initiation of cefiderocol (MIC 0.125 µg/ml) in combination with meropenem resulted in rapid clearance of blood cultures. The patient completed a 6-week course of dual therapy, underwent valve replacement, and continued cefiderocol plus meropenem for an additional six weeks. At >12 months follow-up, the patient remained relapse-free. Analysis of the explanted valve by fluorescence in situ hybridization confirmed the presence of metabolically active bacteria within biofilm, underscoring the contribution of biofilm persistence to therapeutic failure and the critical role of surgical intervention (56).

Taken together, cefiderocol has been administered for a median of 13-14 days in most systemic infections, and up to six weeks in endocarditis or device-related infections. Clinical success rates across case series and observational cohorts generally range from 70 to 80%, though microbiological relapse is common in CF and mortality remains high in severely ill or immunocompromised hosts (27-40%) (18,68). Combination regimens are frequently adopted, reflecting both clinical caution and the potential for resistance emergence.

In conclusion, although current evidence remains limited to case reports, small series, and real-world analyses, cefiderocol represents one of the very few agents with reproducible activity against multidrug-resistant *Achromobacter* spp. Its role as a salvage therapy is supported by consistent *in vitro* and *in vivo* data, as well as clinical outcomes across diverse patient populations. Remaining challenges include long-term eradication in CF, resistance emergence during therapy, and persistently high mortality in vulnerable hosts.

## 6. Conclusions

*A. xylosoxidans* causes a variety of diseases, involving patients with multiple comorbidities, resulting in difficult-to-treat infections with poor outcomes. The strains often present multidrug resistance profiles, reducing the weapons against the bacteria. Cefiderocol is a promising option that can be a primary choice for treating *A. xylosoxidans* infections. The problems concerning the precise methods for determining the true susceptibility to this molecule should not hinder its clinical applicability, as demonstrated by its real-life uses, even when discrepancies between *in vitro* and *in vivo* data occur, favouring the latter. This review provides an overview of the impact of *A. xylosoxidans* in clinical practice, its resistance mechanisms, and the potential for better use of cefiderocol as an effective treatment.

## Figures and Tables

**Table I:** Published clinical reports and observational studies on cefiderocol use for *Achromobacter xylosoxidans* infections.

First author, year	Study design/population	Type of infection	Clinical outcome	(Refs.)
Bodro *et al*, 2021	Case report (n=2)	Device-related infection (LVAD-associated infection due to *A. xylosoxidans*)	Clinical resolution without device removal following combination therapy (cefiderocol + tigecycline + piperacillin-tazobactam)	(69)
La Bella *et al*, 2021	Case report	Aortic endocarditis in a patient with non-Hodgkin lymphoma	Clinical cure with cefiderocol-based combination therapy; increased serum bactericidal activity documented	(70)
Warner *et al*, 2021	Compassionate-use case series (n=8; 12 treatment courses)	Pulmonary infections in CF	Clinical response in 91.6% of episodes; microbiological relapse in 91.6% within 180 days	(71)
Gainey *et al*, 2020	Case report (paediatric)	Chronic pulmonary infection in CF	Rapid clinical improvement and sustained microbiological suppression with cefiderocol plus bacteriophage therapy	(72)
Torre-Cisneros *et al*, 2025	Early access real-world cohort (n=5 *Achromobacter* cases)	Respiratory, urinary tract, and catheter-related bloodstream infections	Clinical cure in 80%; 28-day all-cause mortality 40%	(18)
Kirkegaard-Biosca *et al*, 2024	Retrospective observational study (n=5 *Achromobacter* cases)	Systemic infections due to VIM-producing strains	Clinical failure at day 14: 15%; 30-day mortality: 27%; resistance emergence observed in one case	(68)
Wackernagel *et al*, 2025	Case report	Prosthetic valve endocarditis after TAVI	Sustained clinical and microbiological cure after prolonged cefiderocol-based combination therapy and valve replacement	(56)

CF, cystic fibrosis; LVAD, left ventricular assist device; TAVI, transcatheter aortic valve implantation.

## Data Availability

Not applicable.
